# Extranodal natural killer/T-cell lymphoma nasal type with central nervous system involvement mimicked tuberculous meningitis: A case report

**DOI:** 10.1097/MD.0000000000016747

**Published:** 2019-08-23

**Authors:** Yina Yang, Zhouling Li, Chen Zhiyang, Hui Liang

**Affiliations:** a Department of Neurology, Ninghai First Hosptial, Zhejiang; b Department of Neurology, First Affiliated Hospital, School of Medicine, Zhejiang University, Hangzhou, China.

**Keywords:** extranodal NK/T-cell lymphoma, metastasis, misdiagnosis, tuberculous meningitis

## Abstract

**Rationale:**

Neurologic deficits are rare in patients with extranodal natural killer/T-cell lymphoma (NKTL), nasal type. We present a case that was initially suspected as tuberculous meningitis, but later diagnosed as central nervous system metastasis of NKTL, nasal type, which has never been published previously.

**Patient concerns:**

A 55-year-old Chinese man presented with persistent headache and fever. The initial head computed tomography and magnetic resonance imaging (MRI) scan was normal. Low glucose, elevated protein, and pleocytosis of cerebral spinal fluid led to a diagnosis of tuberculous meningitis. The patient did not respond to anti-tuberculosis treatment, and his symptoms aggravated. MRI showed abnormal lesions in the right hemisphere and a lesion in the maxillary sinus region.

**Diagnosis:**

Endoscopic biopsy of the maxillary lesion showed features consistent with NKTL. Positron emission tomography revealed a hypermetabolic mass involving the right maxillary sinus and brain.

**Interventions:**

The patient received chemotherapy.

**Outcomes:**

The patient died 30 days after chemotherapy.

**Lessons:**

Lymphoma of the nasal cavity and paranasal sinuses is extremely rare and may be easily misdiagnosed. Nasal NKTL metastasis should be considered when a patient presents with symptoms of leptomeningeal involvement.

## 1 Introduction

Extranodal natural killer/T-cell lymphoma (NKTL), nasal type, is a rare subtype of non-Hodgkin lymphoma that is usually associated with an aggressive clinical course.^[1]^ The tumor often presents intranasally and has been reported to spread to multiple extranodal sites, including the gastrointestinal tract, lung, and skin.^[2,3]^ NKTL invasion or metastasis to the central nervous system (CNS) is uncommon. Here, we present the case of a 55-year-old male patient that was misdiagnosed as tuberculous meningitis initially, and but when clinical improvement did not occur was determined to be NKTL metastasis of the CNS.

## 2 Case report

A 55-year-old Chinese man presented with a 20-day history of persistent headache and fever. One month previously, he had received a diagnosis of rhinosinusitis because of slight right nasal obstruction. The patient had no specific past history. Neurological examination disclosed bilateral papilledema and neck stiffness. Laboratory tests revealed negative results for the following: rapid plasma regain; microhemagglutination assay for *Treponema pallidum*; human immunodeficiency virus; anti-double-stranded DNA antibody; fluorescent antinuclear antibody test; angiotensin-converting enzyme; toxoplasma antibody; circulating anti-neutrophil cytoplasmic antibody; and complete blood count. Chest x-ray and head CT scan were normal. Examination of cerebral spinal fluid (CSF) disclosed a high opening pressure of 250 cmH_2_O, lymphocyte pleocytosis (20/mm^3^), elevated protein concentration (1.35 g/dL), and low glucose level (0.5 mmol/L). Gram stains and CSF culture were negative. The results were suspect of tuberculous meningitis.

**Figure 1:**
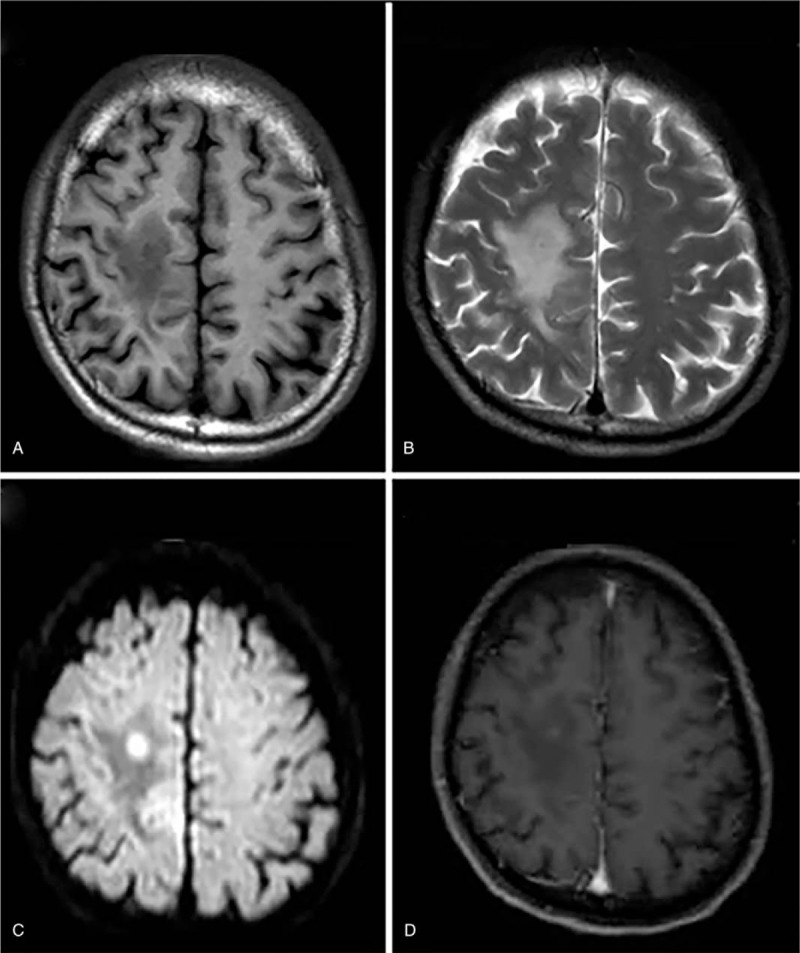
Brain MRI. A, B, T1WI and T2WI showed lesions with peripheral edema involving white matter of right hemisphere. C, D, Diffusion-weighted imaging (DWI) signals were high with mild enhancement. MRI = magnetic resonance imaging.

Anti-tuberculosis therapy was started, including isoniazid, rifampicin, pyrazinamide, ethambutol, and corticosteroids. Fourteen days later, the fever disappeared. Lumbar puncture was repeated and the results were consistent with those previous; no fungi or bacteria were detected in the CSF. Twenty days after admission, the patient presented with tinnitus and left limb weakness. On MRI, there were abnormal lesions in the white matter of the right hemisphere, and the pattern of enhancement was consistent with abscess formation (Fig. 1). Anti-tuberculosis therapy was continued, while left limb weakness further deteriorated.

**Figure 2:**
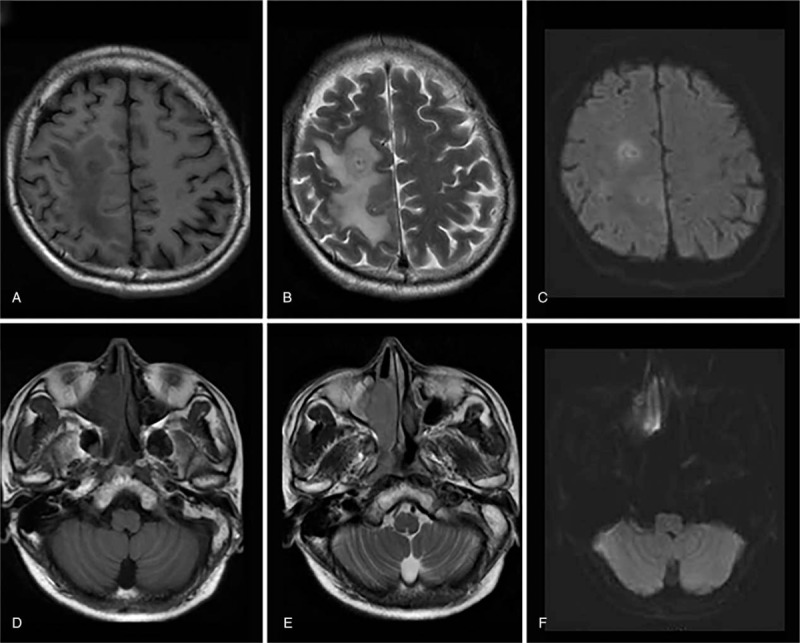
Repeated MRI. A–C, Cranial involvement increased in size. D, E, An isointense mass (arrow) in the region of right maxillary sinus. F, Axial DWI shows restricted diffusion of the lesion.

Thirty days after admission, a focal seizure occurred and repeat MRI showed enlargement of the lobe lesions. Simultaneously, there was a lesion in the region of the right maxillary sinus with restricted diffusion (Fig. 2).

Since clinical improvement was not observed, a diagnostic endoscopic biopsy of the maxillary lesion was performed. Pathologic evaluation showed small atypical lymphoma cells with necrosis, consistent with NKTL. Immunohistochemical staining of the tumor cells was positive for CD3, CD5, and CD56. CD20 and Bcl-2 (B-cell lymphoma 2) testing showed negative results.

**Figure 3:**
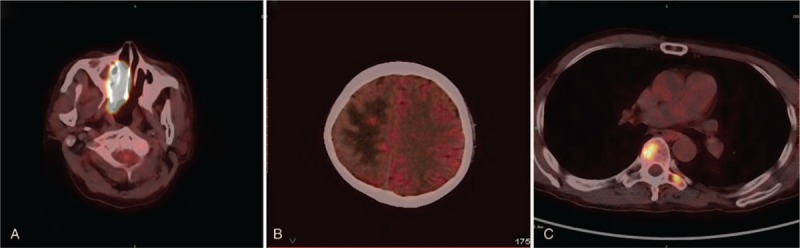
Positron emission tomography CT performed after biopsy revealed a hypermetabolic mass involving the (A) right maxillary sinus, (B) right frontal lobe, and (C) transverse process and T6 vertebra.

Positron emission tomography CT performed after the biopsy revealed a hypermetabolic mass involving the right maxillary sinus and right frontal lobe. In addition, it showed diffuse hypermetabolic activities in the transverse process and T6 vertebra (Fig. 3). These findings supported the diagnosis of NKTL, with extension into the vertebral column and brain. A bone marrow study was performed but did not show any metastatic findings.

The patient was then transferred to the hematology department for further treatment, including chemotherapy (lomustine, methotrexate, vepeside, and dexamethasone). He refused further management and returned home with appropriate medication for discomfort and pain. He died 30 days after chemotherapy, from his rapidly progressive disease.

## 3 Discussion

Extranodal NKTL, nasal type, is a predominantly extranodal malignancy. It is most commonly positive for Epstein-Barr virus and CD56, reflecting a natural killer cell phenotype. Occasional patients lack CD56 expression (i.e., a cytotoxic T-cell phenotype).^[4]^ This tumor is thought to derive from the malignant transformation of mature NK cells or post-thymic T cells.^[5]^ Extranodal NKTL, nasal type, accounts for 1% of all lymphomas in patients of European descent, and the incidence is higher in Asian, South American, and Hispanic peoples.^[2]^ NKTL is an aggressive extranodal tumor with often rapid and fatal evolution.^[4]^ A favorable outcome is only achieved with early diagnosis and treatment, but it is not always easy to recognize NKTL.

NKTL is usually in the nasal cavity or paranasal sinuses, at which location it was previously called idiopathic midline destructive lesion or angiocentric lymphoma. Alternatively, in cases in which cells with angiocentric orientation appeared to form granulomas, it was known as malignant midline granuloma or lethal midline granuloma-NKTL.^[5,6]^

NKTL typically is first encountered with midline destruction, septal perforation, and necrosis of the surrounding tissue. Affected patients present with nonspecific rhinitis or refractory chronic sinusitis and describe typical associated symptomatology. The patient's nonspecific initial complaints generally lead to additional medical investigations, resulting in an accurate diagnosis only after multiple clinic visits. With disease progression, additional conditions develop, including epistaxis, palatal destruction, orbital swelling with erythema, and surrounding facial edema and pain. Initially, symptoms may be localized, but as the disease progresses, they may intensify and become systemic and generalized to include weight loss, malaise, and fever. CT and MRI may indicate an extensive soft tissue mass obliterating the nasal passages and surrounding sinuses, with additional involvement and erosion of adjacent alveolar bone, hard palate, or orbits. After the diagnosis is determined, imaging during clinical follow-ups may help to monitor disease progression or response to treatment.

Neurologic deficits are rare in patients with nasal NKTL. Luther et al^[7]^ reported that fewer than 3% of cases of NKTL were associated with intracranial involvement. However, CNS involvement always occurs concomitantly with locally destructive nasal tumor, or with recurrent and widespread disease.^[8]^ In some cases, NKTL reached the CNS through cranial nerves.^[9]^

When intracranial extension does occur, the process usually is characterized by an abscess-like appearance on neuroimaging, as seen in our patient. This appearance is caused by the angiocentric growth pattern and surrounding destruction, resulting in zonal necrosis. If nasal NKTL cases involve invasion or metastasis to meninges, patients may experience visual disturbances and cranial nerve palsies.^[7]^

In our present case, low glucose, elevated protein, and pleocytosis of the CSF led to the initial diagnosis of tuberculous meningitis. However, the lack of response to anti-tuberculosis treatment and the progress of the disease excluded this.

Some reasons that contributed to the initial misdiagnosis are as follows. First, the initial symptoms of nasal lymphoma are subtle. Patients tend to ignore their symptoms until they become serious; Yen et al^[10]^ found that the average time between patients’ awareness of their sinonasal symptoms and their decision to seek medical help was 8.9 months. Second, the patient was initially thought to have tuberculous meningitis because of the characteristic clinical and CSF profiles. This case reflects the considerable diagnostic difficulty in identifying the cause of chronic meningitis with a persistently low CSF glucose, when standard microbiological and cytological investigations are negative. While CSF cytology is essential for the diagnosis of CNS hematologic malignancies, the cytomorphology of some patients has been negative.^[11]^

Because of the rarity of nasal NKTL, treatment recommendations are based primarily on the results of prospective cohort reports. In comparison with other T-cell and B-cell lymphomas, this disease tends to be quite aggressive and has a poor prognosis.^[2]^ The recommended treatment for NK/T-cell lymphoma is a combination of anthracycline-based CHOP chemotherapy (cyclophosphamide, doxorubicin, vincristine, and prednisone) with local radiotherapy.

Multiple studies have attempted to ascertain the most successful treatment regimen. However, because of the rarity of this disease and the small sample size of each report, the optimal treatment remains controversial. For example, a series of 79 cases of early stage disease treated by radiation therapy alone, or combined with other modalities, showed overall poor prognosis. The 5-year disease-free and overall survival rates were only 35.5% and 37.9%, respectively.^[2]^

CNS extension is a sign of poor prognosis. In 1 study, 5 patients with CNS involvement received CHOP treatment and radiotherapy. All the patients died within the first 6 months after diagnosis.^[9]^

In conclusion, lymphoma of the nasal cavity and paranasal sinuses is extremely rare and may mimic benign processes. Nasal NKTL metastasis should be kept in mind when patients present with nonspecific neurologic symptoms with cranial or spinal leptomeningeal involvement.

## Acknowledgments

The authors thank the family members of the patient for their help and informed consent for publication of the case.

## Author contributions

**Conceptualization:** Yina Yang.

**Data curation:** Yina Yang.

**Formal analysis:** zhouling li.

**Investigation:** Chen Zhiyang.

**Supervision:** hui liang.

**Writing – original draft:** Yina Yang.
